# What is Aging?

**DOI:** 10.3389/fgene.2012.00134

**Published:** 2012-07-20

**Authors:** Michael R. Rose, Thomas Flatt, Joseph L. Graves, Lee F. Greer, Daniel E. Martinez, Margarida Matos, Laurence D. Mueller, Robert J. Shmookler Reis, Parvin Shahrestani

**Affiliations:** ^1^Department of Ecology and Evolutionary Biology, University of CaliforniaIrvine, CA, USA; ^2^Department of Biomedical Sciences, Institute of Population Genetics, Vetmeduni ViennaVienna, Austria; ^3^Joint School of Nanoscience and Nanoengineering, North Carolina A&T State University, UNC GreensboroGreensboro, NC, USA; ^4^Department of Biology, La Sierra UniversityRiverside, CA, USA; ^5^Department of Biology, Pomona CollegeClaremont, CA, USA; ^6^Centro de Biologia Ambiental, Departamento de Biologia Animal, Faculdade de Ciencias da Universidade de LisboaCampo Grande, Lisboa, Portugal; ^7^Central Arkansas Veterans Healthcare System, Department of Geriatrics and Biochemistry/Molecular Biology, University of Arkansas for Medical SciencesLittle Rock, AR, USA

In 1991, the book *Evolutionary Biology of Aging* offered the following definition of aging: *a persistent decline in the age-specific fitness components of an organism due to internal physiological deterioration* (Rose, 1991). This definition has since been used by others a number of times. However, it was only a modest generalization of a definition proffered by Alex Comfort over three editions (1956–1979) of his key book *The Biology of Senescence* (Comfort, 1979): “a progressive increase throughout life, or after a given stadium, in the likelihood that a given individual will die, during the next succeeding unit of time, from randomly distributed causes.” The 1991 definition chiefly added reproductive fitness components to Comfort's definition, while adding the qualifiers that the fitness-component decline should be persistent and should be “due to internal physiological deterioration,” where the latter phrase was meant fairly broadly. Thus increases in mortality with age due to chronic infections such as HIV/AIDS were excluded by the 1991 definition.

Yet a mere definition does not necessarily tell a scientist what causally underlies the phenomenon that is so defined. The latter issue is much broader, implicitly raising fundamental scientific questions regarding mechanisms. As a simple but related example, the term *adaptation* can be defined as an attribute that enhances the net reproduction of members of a particular population or species, and yet many deeper issues are invoked by a question like, “*What is adaptation*?” A creationist, for example, could view adaptation as an attribute that is so defined, yet further assume that all such adaptations are specifically bequeathed to organisms by an omnipotent creator. By contrast, an evolutionary biologist would instead assume that such adaptations are necessarily products of natural selection, acting directly or indirectly.

Before 1992, almost every scientist who thought about aging assumed that it progressed without remit to the point of death. Evolutionary biologists further thought that this inexorable deterioration was brought about by the progressive decline in Hamilton's forces of natural selection (Hamilton, 1966; Rose et al., 2007). With respect to the underlying physiological machinery of aging, the only difference between most evolutionists and most gerontologists at that time was that evolutionists overwhelmingly expected that there were likely to be many physiological mechanisms of deterioration, rather than a few (Williams, 1957; Rose, 1991). Thus the aforementioned 1991 book accommodated commonly inferred physiological mechanisms of aging within an overarching evolutionary framework, thus delineating an “evolutionary biology of aging” that subsumed conventional gerontological thinking, rejecting only those parts that were inconsistent with evolutionary theory.

This synthesis of evolutionary biology and gerontology survived for only 1 year before being undermined by two 1992 papers, Curtsinger et al. (1992) and Carey et al. (1992), in which demographic aging was shown to subside in late-life among cohorts of *Drosophila* and the medfly. Some initial attempts to accommodate their results focused on possible density artifacts (Nusbaum et al., 1993), but a substantive series of experiments from the Curtsinger lab (Khazaeli et al., 1995, 1998a,b) demolished such quibbling. By 1995, it was clear that the cessation of aging was a genuine phenomenon rather than an experimental artifact.

Yet another possibility remained, one that had been discussed in 1939 by Greenwood and Irwin (1939) in their article showing that human aging stopped demographically: lifelong heterogeneity. This is a concept that has been mathematically developed, particularly by Vaupel (Vaupel et al., 1979; Vaupel, 1988), but it is fairly easy to convey as a verbal argument. If a cohort consists of sub-cohorts that differ radically in their lifelong robustness, then the less robust will be eliminated early, leaving only the much more robust individuals. If these surviving sub-cohorts are robust enough, demographic aging should greatly slow at very late ages.

Many have looked for evidence of such an association between lifelong robustness and the cessation of aging (Khazaeli et al., 1998a,b; Drapeau et al., 2000; Mueller et al., 2003; Rauser et al., 2005). It *is* possible to produce such a mortality-rate flattening by artificially constructing cohorts out of very different sub-cohorts (Brooks et al., 1994), but no one has yet found enough naturally occurring lifelong heterogeneity to generate demographic plateaus in age-specific mortality or fecundity (reviewed in Shahrestani et al., 2009; Mueller et al., 2011). Indeed, there are good evolutionary genetic reasons to expect that such lifelong heterogeneity will rarely arise: natural selection will oppose the maintenance of such heterogeneity, whether it is due to genetic polymorphism or extreme non-genetic plasticity (Mueller et al., 2011, Chapter 7). Natural selection instead favors the maintenance of genetic variation affecting fitness-components when that genetic variation has opposed effects at different ages, in other words antagonistic pleiotropy, not lifelong effects that are consistent in direction (Rose, 1985; Mueller et al., 2011).

Thus it appears that the cessation of aging occurs at the individual level, and is not just an artifact of population structure. Yet this is clearly paradoxical, if we think of the machinery of aging in terms of such physiological processes as steadily cumulative damage. If it is supposed that some process of cumulative damage or disharmony is supposed to underlie aging, why should that process abruptly stop at the very point, late in adult life, when it has greatly reduced the ability of the surviving individuals to sustain life and reproduction?

Mueller, Rauser, and Rose instead developed very different models for the evolution of late-life plateaus in mortality and fecundity (Mueller and Rose, 1996; Mueller et al., 2011), using the eventual plateaus in Hamilton's forces of natural selection as their core explanatory principle for mortality and fecundity plateaus late in adult life. These formal mathematical models, founded in evolutionary genetics, show that it is perfectly reasonable for natural selection to produce late-life plateaus in life-history characters, especially with finite population sizes, once the forces of natural selection have fallen to very low values. Of greater significance for strong-inference science, they further demonstrated that experimental evolution can tune the timing of the cessation of *Drosophila* aging in conformity with these theoretical results (Rose et al., 2002; Rauser et al., 2006). Indeed, the discovery that aging stops turns out to be a powerful corroboration of Hamilton's original results for the forces of natural selection (Hamilton, 1966; Mueller et al., 2011), all the more dramatic because it was counterintuitive and hence unexpected.

These results call for some fundamental re-thinking of what aging is. Twenty years ago, evolutionary biologists imagined that once Hamilton's forces of natural selection reached zero, death should quickly follow due to the absence of natural selection opposing the effects of cumulative damage and/or regulatory disharmony. Now at least some have a very different vision (Mueller et al., 2011). As Hamilton's forces of natural selection decline during the first part of adulthood, we might say that age-specific elements of adaptation are de-tuned. This de-tuning in turn could be said to generate the demographic phenomena of aging, as well as the myriad physiological dysfunctions that we know as the seemingly, but actually secondary, mechanistic foundations of aging. In species with sufficiently severe antagonistic pleiotropy between reproduction and adult survival, such as Pacific salmon, soybean, and mayflies, all members of a cohort may die without either a well-defined period of aging or late life, in the absence of human intervention. But under sufficiently benign environmental conditions, individuals from species as disparate as humans and fruit flies can survive a protracted aging period and reach a subsequent late-life respite in which fitness-component deterioration stops, a phase permitted by the complete attenuation of the forces of natural selection relative to the effects of genetic drift.

The above results suggest that aging is *not* inevitably a cumulative and unremitting process of deterioration. Instead, aging might be best conceived as a facet of adaptation, specifically its de-tuning during the first part of adulthood. This de-tuning is due to the steady declines in the forces of natural selection that occur after the start of adulthood in most populations. Once those declines stop, aging eventually ceases, and adaptation stabilizes albeit at a low level. There is little sign of a physiological “momentum” that necessarily advances aging until every member of a cohort has died; nor is there any *a priori* requirement for such constancy, despite the seductive analogy to Newtonian physics. An important corollary is that many of the standard biological intuitions about aging, particularly those that associate it with the Second Law of Thermodynamics, are not generally valid. Some functional declines of physiological characters continue into late-life, and some even accelerate, whereas other functional declines come to a halt (Shahrestani et al., 2012). There is thus no scientific justification for assuming that each and every type of physiological deterioration that has been associated with aging must continue without remit throughout late adult life.

This realization leads to another fundamental change in our thinking about “the process of aging”: it is not actually a physiological *process*, in and of itself. Although it certainly involves physiological changes, the physiology of aging is molded and constrained according to the dictates of natural selection shaping adaptation. Some of the genetic foundations of adaptation serve to sustain survival and reproduction later in life, presumably because of age-independent benefits (Charlesworth, 2001). Other features of adaptation are apparently subject to age-specific and pleiotropic genetic effects which undermine age-specific mortality and fecundity, together with their underlying physiology, during middle adulthood (Rose, 1991; Rose et al., 2002; Mueller et al., 2011). In extreme cases of trade-offs between survival and reproduction, continued adult survival may be wholly sacrificed by natural selection, resulting in semelparous, univoltine, or annual life cycles (Rose, 1991). All these possibilities for patterns of aging are permitted by evolution.

The evolutionary biology of aging proposed in 1991 (Rose, 1991) provided some warrant for allowing gerontologists to conduct their research largely without evolutionary considerations. The falling forces of natural selection were supposed to ensure the cumulative and unremitting physiological deterioration commonly assumed by gerontologists. But now neither that evolutionary rationale nor that type of mechanistic thinking seem warranted, given what we know of the cessation of aging. At its very foundations, aging is a multifaceted phenomenon that is a derivative feature of the evolutionary biology of adaptation, *not a single* physiological process, even though adaptations generally involve physiology.

As such, aging is best studied in light of the methodological strictures and theoretical scaffolding supplied by evolutionary biology. Some of those elements were sketched in 1991 (Rose, 1991), but the analysis offered then was far too simplistic. We now know that aging is much more complex than was understood then, both genomically (Rose and Burke, 2011) and demographically (Mueller et al., 2011), and it is inseparable from adaptation itself (Rose, 2009). This makes it a hazardous proposition to study aging without significant attention to evolutionary genetics. An evolutionary-genetic perspective on aging raises several points of concern, including the difficulty of studying aging under conditions in which adaptation has been undermined or distorted, such as breeding regimes that create inbreeding depression, highly artificial genotype-by-environment interactions, and obscure evolutionary history (Rose et al., 2011). As aging is neither more nor less than the deterioration of adaptation with adult age, obscuring the features of adaptation by performing experiments with laboratory cohorts of an abnormally inbred and/or mutated strain with a poorly documented history of laboratory culture has created and will perpetuate significant difficulties of interpretation.

This vision of what underlies aging may be off-putting for some, given its theoretical complexities and difficulties for experimental design. No doubt many physicists felt the same way about the destruction of the elegant late nineteenth Century version of Newtonian mechanics by the advent of relativistic and quantum mechanics, in the period from 1905 to 1945. But paradigm transitions in science are generally like that, requiring that we abandon comfortable theories in favor of those that are significantly less wrong.

The genetics of aging cannot go on as it did before 1992. We need not jettison every lesson gleaned from past research, whether evolutionary or mechanistic, though conclusions reached under the quondam paradigm now require re-examination within our current, broader understanding. We will be able to salvage those parts that can be reintegrated within a scientific framework for the evolutionary genetics of aging, developed in light of its fundamental nature: de-tuned adaptation during the first part of adulthood. But a new evolutionary genetics of aging must now be built.
